# New Insights in *Acanthamoeba*

**DOI:** 10.3390/pathogens11050609

**Published:** 2022-05-23

**Authors:** María Reyes-Batlle, Ines Sifaoui, Rubén L. Rodríguez-Expósito, José E. Piñero, Jacob Lorenzo-Morales

**Affiliations:** 1 Instituto Universitario de Enfermedades Tropicales y Salud Pública de Canarias, Universidad de La Laguna (ULL), 38206 Tenerife, Spain; isifaoui@ull.edu.es (I.S.); rrodrige@ull.edu.es (R.L.R.-E.); jpinero@ull.edu.es (J.E.P.); jmlorenz@ull.edu.es (J.L.-M.); 2 Departamento de Obstetricia, Ginecología, Pediatría, Medicina Preventiva y Salud Pública, Toxicología, Medicina Legal y Forense y Parasitología, Universidad De La Laguna, La Laguna, 38203 Tenerife, Islas Canarias, Spain; 3 Red de Investigación Cooperativa en Enfermedades Tropicales (RICET), 28029 Madrid, Spain; 4 CIBER de Enfermedades Infecciosas (CIBERINFEC), Instituto de Salud Carlos III, 28029 Madrid, Spain

*Acanthamoeba* is a free-living amoeba genus able to cause severe infections, such as Granulomatous amoebic encephalitis (GAE), epithelial disorders and a sight-threatening disease called *Acanthamoeba* keratitis (AK) [1,2]. These opportunistic pathogenic protozoa have been isolated from different sources, with preference for soil and water niches [3], and they can be classified into 23 genotypes based on their 18S rRNA sequences [4]. Moreover, these organisms are capable of surviving in both external environments as well as inside an infected host as parasites. This double condition causes them to be called amphizoic organisms.

The genus *Acanthamoeba* is considered an emerging parasite due to the difficulty of treating infections by these organisms [5]. Furthermore, its clinical importance not only falls on its capability to produce infections, but also on its ability to harbor other pathogenic microorganisms as endosymbionts [6]. The wide distribution of *Acanthamoeba* in natural environments such as soil, airborne dust and water and the underdiagnosis of GAE and AK cases have made it difficult to investigate possible sources of infection. Moreover, the lack of a stable classification of this genus complicates the identification of individual isolates, including the matching of amoebae from infections with organisms from the environment. 

*Acanthamoeba* is able to produce a life-threatening central nervous system (CNS) infection among the immunocompromised population, known as Granulomatous amoebic encephalitis (GAE). Although the prevalence of GAE worldwide is low, the mortality rate oscillates between 95 and 98% among infected patients [7]. On the other hand, the most common pathology due to *Acanthamoeba* is *Acanthamoeba* keratitis (AK), produced in healthy individuals, especially contact lens wearers [2]. Lately, the number of AK infections has been increasing from 17 to 70 per million among contact lens wearers [8]. The contamination of contact lens cases with amoebae is the first step in AK infection [8]. Generally, contact lens solutions include anti-fungal and anti-microbial components, which are not fully effective against *Acanthamoeba* trophozoites and cysts [9]. On the other hand, the current AK treatments are normally toxic, last a long time and are non-specific [2]. Therefore, the development of new formulations against *Acanthamoeba* strains are needed.

*Acanthamoeba* detection methods are mainly based on microscopic techniques, but these methods are laborious, long-lasting and require amoeba culture [2]. In addition, amoeba culture is only positive in around 55% of the cases, and the quantity of the obtained sample could be not enough to see the protozoa. PCR (polymerase chain reaction) testing is a rapid detection technique of *Acanthamoeba* parasites at the genotypic-level (T1-T23) [10]. *Acanthamoeba* genotyping is gaining importance at the treatment level because different genotypes show variation in symptoms and response to the treatment [11]. However, PCR presents different limitations, such as insufficient target DNA yield or non-specific DNA amplification [12]. Thus, in order to reduce the AK infection damage, new rapid and reliable diagnostic tools need to be developed.

The current *Pathogens* Journal Special Issue, New Insights in *Acanthamoeba*, is focused on the most recent advances related to the pathogenesis, diagnosis and treatment of this opportunistic parasite. All these improvements mean significant and laborious research, which contributes to establishing further treatments and diagnosis tools, as well as to better understanding *Acanthamoeba* pathogenesis. From the *Pathogens* Journal and as Guest Editors, we would like to acknowledge the authors for their contributions and encourage them to continue with their interesting investigations.

## References

[1] Siddiqui R., Khan N.A. (2012). Biology and pathogenesis of Acanthamoeba. Parasites Vectors.

[2] Lorenzo-Morales J., Khan N.A., Walochnik J. (2015). An update on Acanthamoeba keratitis: Diagnosis, pathogenesis and treatment. Parasite.

[3] Schuster F.L., Visvesvara G.S. (2004). Free-living amoebae as opportunistic and non-opportunistic pathogens of humans and animals. Int. J. Parasitol..

[4] Putaporntip C., Kuamsab N., Nuprasert W., Rojrung R., Pattanawong U., Tia T., Yanmanee S., Jongwutiwes S. (2021). Analysis of Acanthamoeba genotypes from public freshwater sources in Thailand reveals a new genotype, T23 Acanthamoeba bangkokensis sp. nov. Sci. Rep..

[5] Khan N.A. (2006). Acanthamoeba: Biology and increasing importance in human health. FEMS Microbiol. Rev..

[6] Iovieno A., Ledee D.R., Miller D., Alfonso E.C. (2010). Detection of Bacterial Endosymbionts in Clinical Acanthamoeba Isolates. Ophthalmology.

[7] Martinez A.J., Visvesvara G.S. (1997). Free-living, amphizoic and opportunistic amebas. Brain Pathol..

[8] Lorenzo-Morales J., Martín-Navarro C.M., López-Arencibia A., Arnalich-Montiel F., Piñero J.E., Valladares B. (2013). Acanthamoeba keratitis: An emerging disease gathering importance worldwide?. Trends Parasitol..

[9] Padzik M., Hendiger E.B., Żochowska A., Szczepaniak J., Baltaza W., Pietruczuk-Padzik A., Olędzka G., Chomicz L. (2019). Evaluation of in vitro effect of selected contact lens solutions conjugated with nanoparticles in terms of preventive approach to public health risk generated by Acanthamoeba strains. Ann. Agric. Environ. Med..

[10] Bhosale N.K., Mandal J., Parija S., Ahuja S. (2016). Utility of Polymerase chain reaction in diagnosis of Acanthamoeba and Microsporidial keratitis. Int. J. Infect. Dis..

[11] Arnalich-Montiel F., Lumbreras-Fernández B., Martín-Navarro C.M., Valladares B., Lopez-Velez R., Morcillo-Laiz R., Lorenzo-Morales J. (2014). Influence of Acanthamoeba genotype on clinical course and outcomes for patients with Acanthamoeba keratitis in Spain. J. Clin. Microbiol..

[12] Rasheed A.K., Siddiqui R., Ahmed S.M.K., Gabriel S., Jalal M.Z., John A., Khan N.A. (2020). hBN Nanoparticle-Assisted Rapid Thermal Cycling for the Detection of Acanthamoeba. Pathogens.

